# The Nuclear Lamina: Protein Accumulation and Disease

**DOI:** 10.3390/biomedicines8070188

**Published:** 2020-07-01

**Authors:** Carla Almendáriz-Palacios, Zoe E. Gillespie, Matthew Janzen, Valeria Martinez, Joanna M. Bridger, Troy A. A. Harkness, Darrell D. Mousseau, Christopher H. Eskiw

**Affiliations:** 1 Department of Food and Bioproduct Sciences, University of Saskatchewan, Saskatoon, SK S7N 5A8, Canada; caa806@mail.usask.ca (C.A.-P.); vcm199@mail.usask.ca (V.M.); 2 Department of Biochemistry, Microbiology and Immunology, University of Saskatchewan, Saskatoon, SK S7N 5A8, Canada; zoe.gillespie@usask.ca (Z.E.G.); maj155@mail.usask.ca (M.J.); troy.harkness@usask.ca (T.A.A.H.); 3 Centre for Genome Engineering and Maintenance, College of Health, Life and Medical Sciences, Brunel University London, Kingston Lane, Uxbridge UB8 3PH, UK; joanna.bridger@brunel.ac.uk; 4 Cell Signalling Laboratory, Department of Psychiatry, University of Saskatchewan, Saskatoon, SK S7N 5A5, Canada; darrell.mousseau@usask.ca

**Keywords:** lamina, protein accumulation, premature aging, neurodegeneration, autophagy, clearance

## Abstract

Cellular health is reliant on proteostasis—the maintenance of protein levels regulated through multiple pathways modulating protein synthesis, degradation and clearance. Loss of proteostasis results in serious disease and is associated with aging. One proteinaceous structure underlying the nuclear envelope—the nuclear lamina—coordinates essential processes including DNA repair, genome organization and epigenetic and transcriptional regulation. Loss of proteostasis within the nuclear lamina results in the accumulation of proteins, disrupting these essential functions, either via direct interactions of protein aggregates within the lamina or by altering systems that maintain lamina structure. Here we discuss the links between proteostasis and disease of the nuclear lamina, as well as how manipulating specific proteostatic pathways involved in protein clearance could improve cellular health and prevent/reverse disease.

## 1. Introduction:

The continuous turnover of cellular proteins is vital for the maintenance of cell and organismal health. The processes involved in the maintenance of cellular protein composition can be cumulatively referred to as proteostasis. Disruption of proteostasis can result in the progressive accumulation of insoluble protein aggregates, contributing to disease. For example, in the brain this disruption results in neurodegenerative disorders, including Huntington’s disease, Alzheimer’s disease and Parkinson’s disease [1,2,3] as well as prion diseases [4] (such as Kuru [5] or its variant, Creutzfeldt–Jakob disease [6]). Accumulation of insoluble protein aggregates is not the sole way in which proteostasis is disrupted; indeed, failure of pathways responsible for the maintenance of proteins at physiological levels also results in disruption of cellular function. Under normal, healthy conditions, excess cellular proteins are cleared by several mechanisms, including lysosomal degradation, 26S proteasome (e.g., the ubiquitin–proteasome pathway) and autophagy. Notably, the efficiency of these mechanisms decrease with age. For example, in aged yeast, loss of acidic compartments, such as lysosomes, results in downregulation of ATPases responsible for protein degradation [7,8,9]. Upregulation of these *ATPase* genes increases cellular lifespan, likely via reestablishment of proteostasis. Findings such as these indicate that promoting the activity of protein degradation pathways may be a viable strategy to override pro-aging signals, extending healthspan and possibly lifespan.

One subcellular region that is often overlooked in the context of protein accumulation and proteostasis is the nuclear lamina; yet protein accumulation in this region can lead to equally significant disruption of cellular function. Here we will discuss how protein accumulation within the nuclear lamina is associated with major disease and with cellular aging. Furthermore, we will highlight some of the potential mechanisms that can be manipulated to promote increased cellular health through maintenance or reestablishment of proteostasis.

## 2. Basic Structure and Function of the Nuclear Lamina

**Figure 1:**
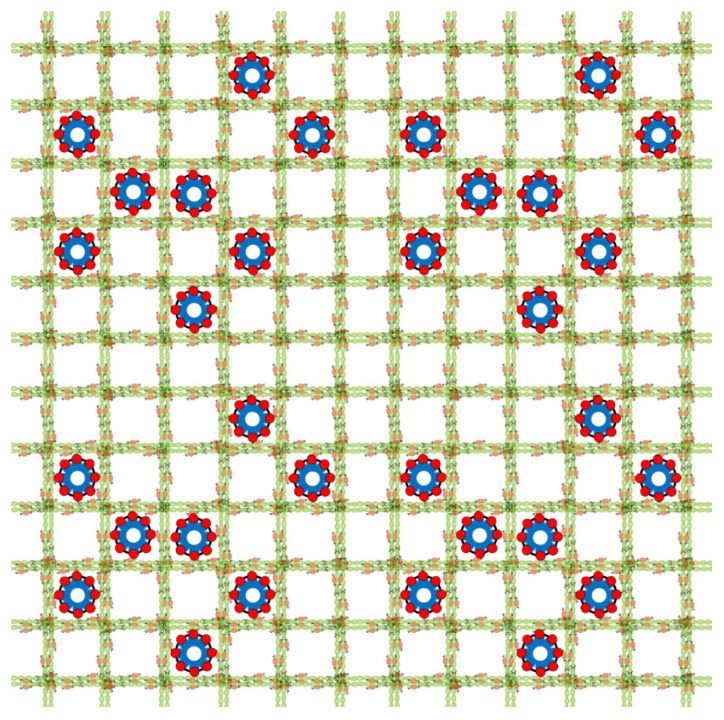
Schematic representation of nuclear lamina structure (as visualized in EM micrographs by Aebi and colleagues [17]). Lamin A/C and Lamin B oligomerize via head-to-tail interactions to form intermediate filaments. These filaments then organize into higher order filaments (green and orange cables) forming a lattice-like pattern under the nuclear envelope that helps to stabilize protein complexes, such as nuclear pore complexes (blue and red structures) and allow for the transport of proteins.

The nuclear lamina is a complex and highly organized meshwork of proteins that covers the nucleoplasmic face of the inner nuclear membrane. Although lower eukaryotes (such as yeast) and embryonic stem cells do not have a conventional nuclear lamina [10,11,12], this structure functions as a platform on which several critical processes are based, in addition to providing structural support to the nucleus [13,14]. The main structural proteins of the lamina are the lamins; type V intermediate filament proteins that have a short N-terminal head, a long central ~45-nm alpha-helical coiled-coil rod domain and a globular tail-domain [15]. These features enable lamin (LMN) dimers to polymerize into a stable, soluble head-to-tail assembly, which then associates into fibers and higher-ordered lattices [16,17] (schematic representation of this structure presented in Figure 1). In mammals, the nuclear lamin proteins are encoded by three genes: *LMNA* (coding for lamin A and lamin C proteins), *LMNB1* (lamin B1) and *LMNB2* (lamin B2). All three lamins are important for structure and undergo different post-translation modifications. Following translation, the precursor form of LMNA (prelamin A) and the B-type LMNs undergo farnesylation at the cysteine residue of the carboxy-terminal -CaaX motif [18]. LMNC, however, does not contain the -CaaX motif and its carboxyl terminus undergoes no further modifications. Following farnesylation, the three terminal amino acids on A and on B-type LMNs are cleaved by either zinc metalloprotease related to Ste24p (ZMPSTE24; prelamin A) or Ras-converting enzyme 1 (Rce1; LMNB1 and B2) and the alpha carboxyl group methylated by isoprenylcysteine carboxyl methyltransferase [19]. Unique to prelamin A, an additional proteolytic cleavage step by ZMPSTE24 occurs 14 amino acids upstream of the farnesylated cysteine, producing mature LMNA. The reasons for some of these different post-translational modifications of lamins are unknown, but the maintenance of farnesylation in B-type LMNs indicates that these proteins are more closely associated with the inner nuclear membrane than LMNA [20]. The A and B-type LMNs further exhibit limited co-localization, with A-type LMNs demonstrating greater nuclear mobility than B-type LMNs [21]. This supports the hypothesis that these proteins have different functions within the lamina structure.

**Figure 2:**
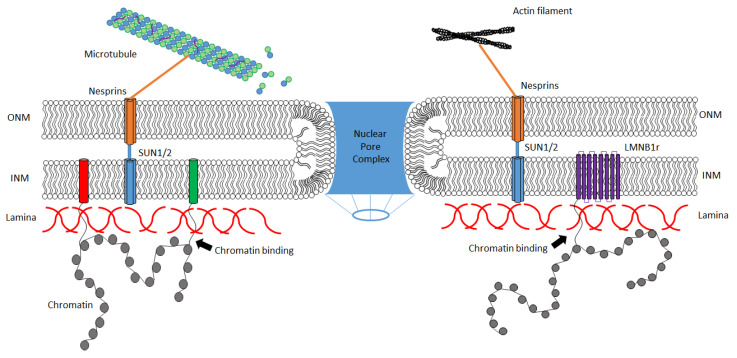
**Schematic representation of lamina interactions.** The nuclear lamina (red wavy lines) is the meshwork under the inner and outer nuclear membranes (INM and ONM, respectively). The lamina has gaps to permit the placement of nuclear pore complexes (blue). INM proteins, such as nuclear envelope transmembrane (NET) and other lamina-associated proteins (red and green cylinders) penetrate through the lamina to make contact with chromatin (gray spheres). Linker of nucleoplasm to cytoplasm complexes (LINC) formed from nesprins and SAD1/UNC-84 homology (SUN) proteins (orange and blue cylinders, respectively) make contact with actin filaments and microtubules.

Whereas the LMNs are the major structural components of the nuclear lamina, other proteins are important for linking the lamina with specific functions (Figure 2). One class of proteins is the nuclear envelope transmembrane proteins (NETs), including lamina associated peptide (LAP) 1, LAP2a, LMNB receptor (LBR) and emerin. The importance of these proteins is underscored by the association between mutations in the genes encoding these proteins and a number of myopathies, lipodystrophies and neuropathies [22,23,24,25,26]. NETS are integral proteins of the inner nuclear membrane and make contact with nucleoplasmic proteins and chromatin. These interactions with chromatin have putative roles in genome organization, with specific NETs interacting with regions of the genome called lamina associated domains (LADs), which are comprised of locally folded/physically organized regions of chromatin. Approximately 40% of the genome is organized into ~1300 LADs [27,28,29,30,31,32,33,34,35]. LADs and other locally folded regions of chromatin (called topologically associated domains—TADs) allow for compartmentalization of important processes such as heterochromatin maintenance, DNA repair and transcription [36,37,38,39]. LADs exhibit cell-type specificity, indicating that changes in NET protein composition in the lamina promotes tissue-specific gene regulation [40]. Associations between LADs and the lamina further facilitate the deposition of epigenetic marks (such as H3K9me2/3 [41]), targeting of epigenetic regulators (H3K9 methyltransferases [42]) and the ability of specific transcription factors and co-regulators to find their targets (such as the LBR [43], cKROX, YY1, HDAC3 [39,44,45] and LAP2B [39,46]). In addition, interaction of chromatin with the lamina likely mediates the positioning of whole chromosomes at the nuclear periphery [47,48,49,50,51,52].

The nuclear lamina, in addition to the nuclear envelope, is also home to the linker of nucleoplasm to cytoplasm complex (LINC) that connects the cytoskeleton to the nucleus. This complex is composed of different splice isoforms of the nuclear envelope spectrin-repeat proteins (nesprins), which contain Klarsicht/ANC1/Syne-1 homology (KASH) domains. Nesprins trimerize and interact with a trimer of SAD1/UNC-84 homology (SUN)-domain containing proteins, with SUN1 being the primary interacting partner [53,54,55,56,57,58]. The LINC-mediated interaction between the cytoskeleton and nuclear protein complexes helps to position the nucleus within cells [59,60,61,62] likely facilitating the transmission of cellular mechanical stress signals and aiding the trafficking of cargo to and from the nucleus [63]. LINC complexes in the nuclear envelope have been directly implicated in actin dynamics by regulating RhoA signaling [64], a known upstream component of the actin signaling cascade. SUN1 interacts with both lamins and nesprins, thus implicating actin- and microtubule-dependent processes in nucleo–cytoplasmic transport [63]. In addition, LMNA/C and emerin have been shown to influence gene expression through a change in nuclear and cytoskeletal actin dynamics, independent of general import and export [65], thus indicating a functional relationship between gene expression and lamina/nuclear envelop tethering to the actin cytoskeleton. The physiological relevance of cytoskeleton interactions with lamina proteins is highlighted by the variety of diseases in which these relationships are disrupted, with a major example being HGPS.

## 3. Hutchinson–Gilford Progeria Syndrome (HGPS): A Protein Accumulation Disease of the Nuclear Lamina?

HGPS is a rare genetic disorder that causes children to age eight times faster than normal. Children with this disease usually succumb to heart failure or stroke by ~14 years of age. HGPS patients exhibit numerous phenotypes of the normal aging process, including osteoporosis, loss of subcutaneous fat, alopecia and atherosclerosis. Although children with this disease die young, the phenotypes are not detected until ~2–3 years of age, indicating that there is a slow progression that is not immediately deleterious. The reason for this initial delay in the emergence of phenotypes is unclear. What is known is that HGPS is caused by a single dominant-negative point mutation (G608G; GGC > GGT) in the *LMNA* gene that activates a cryptic splice site in exon 11 [66,67]. The resulting protein (referred to as progerin) has a 50 amino acid deletion in the C-terminal tail domain that contains the proteolytic cleavage site targeted by ZMPSTE24 critical for producing mature LMNA. This inability to be cleaved leads to the retention of the C-terminal farnesylation, anchoring progerin to the nuclear membrane and resulting in progerin accumulation within the lamina. It has been suggested that the translation rates of progerin increase in HGPS cells, which also contributes to accumulation of proteins and disruption of proteostasis within the lamina [68]. It is well accepted that the accumulation of progerin results in HGPS. While the observed phenotypes are likely the result of increased levels of cellular damage (for example DNA damage), we suggest the hypothesis that the delay in the onset of these phenotypes may indicate that a threshold of progerin accumulation must be reached before disruptions in cellular functions are observed.

**Figure 3:**
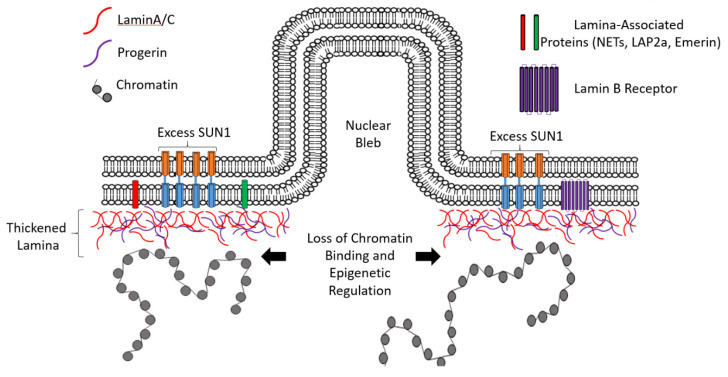
Schematic representation of lamin disruption in Hutchinson–Gilford Progeria Syndrome (HGPS) cells. Nuclear lamina structure becomes thickened with lamin A/C proteins (red lines) in addition to progerin (purple lines). This thickening blocks chromatin (gray spheres) interactions with NETs (red and green cylinders) and Lamin B receptors (purple cylinders). Excess SUN proteins may also accumulate in HGPS cells (blue and orange cylinders). Nuclear blebs form that have large gaps in the lamina structure.

How would accumulation of progerin within the nuclear lamina have such devastating impacts on cells, leading to premature aging? One of the major cellular hallmarks of progerin accumulation is the thickening of the nuclear lamina structure (Figure 3). This thickening not only leads to issues with lamina disassembly during mitosis (leading to mitotic catastrophe) but may also bury chromatin binding and protein–protein interaction domains of lamina-associated proteins within the lamina structure. This hypothesis is supported by observations that whereas HGPS has global impacts across almost all tissues, disruption of specific NET protein function leads to diseases that affect a limited number of tissues [22,69] and does not have the systemic impact of progerin expression. Another consequence of progerin accumulation and lamina thickening is the loss of heterochromatin, LAD structure and gene repression [70,71,72] as a result of lost lamina interactions with epigenetic regulators, such as DNA methyl transferases (DNMT1 [73,74]) and histone methyltransferases (such as EZH2 [71,75]). Chojnowski and colleagues propose that the initial loss of heterochromatin due to progerin accumulation [76] predisposes HGPS cells to other downstream defects such as DNA damage while removal of progerin results in heterochromatin reformation.

These observations lead to a broader question: can the accumulation of wildtype proteins through disruption of protein homeostasis within the lamina be a global mechanism of cellular dysfunction across multiple tissues, driving aging and disease? There is evidence to support this. The nuclear lamins are not expressed equally in all tissues. For example, brain/neuronal tissues have very low levels of LMNA (due to the brain-specific micro RNA-9 (mir-9) preventing pre-lamin A processing) [77] but have higher levels of LMNC [78]. The lower LMNA to LMNC ratio in the brain (compared to somatic tissues) is likely a main contributing factor as to why cognitive perturbations are not observed in HGPS children who would normally express the toxic progerin/*LMNA* splice variant. Mice expressing farnesylated versions of prelamin A in neuronal tissues exhibited esophageal achalasia and abnormal enteric neurons [79], indicating that inappropriate levels of normal lamina proteins can be disruptive, even to post-mitotic cells. Other cell types (HEK293, NHDF and HeLa) overexpressing wild-type LMNA exhibited lattice-like aggregates of the protein and redistribution of LMNC [80]. Overexpression of LMNC in otherwise healthy *Drosophila melanogaster* is stage-specific lethal [16] while overexpression of LMNB1 in HEK293 and neuronal cells results in nuclear stiffness and a phenotype of autosomal-dominant leukodystrophy (ADLD) [81]. In contrast, neurons in the brains of the Alzheimer patients have significant reductions in LMNB levels which leads to the formation of invaginations of the nuclear lamina into the deep nuclear interior and mechanistically important for disease [82,83]. These examples highlight the importance of maintaining the correct balance of LMN proteins within the lamina structure for normal cellular function.

The SUN1 protein has also been observed to accumulate concomitantly with progerin in the lamina in HGPS cells [84], with progerin further interfering with SUN1 function in the LINC [85]. Knockdown of SUN1 by siRNA ameliorated the abnormal phenotypes observed in HGPS, improving nuclear morphology, increasing heterochromatin levels and decreasing levels of senescence [86]. Therefore, it is possible that the accumulation of progerin promotes the buildup of other lamina-associated proteins, thereby compounding cellular defects. Furthermore, the accumulation of the incorrect ratio of splice isoforms may also have an impact on lamina structure and function. There are 10 predicted splice variants of SUN1 (not all of which have been detected), with all variation occurring in the N-terminal nucleoplasmic DNA binding domain [53,87,88]. There is limited information on the specific functions of these variants and it has been shown that HGPS cells express a previously uncharacterized isoform of SUN1 which may be responsible for the observed genomic instability and disruption of chromosome positioning within the nuclear volume of HGPS cells [89,90]. Although the mechanisms controlling the expression of these variants are still unknown, the accumulation of different isoforms may have functional consequences. In addition to this, it is possible that the premature aging phenotypes associated with HGPS may not result from the function of any specific SUN protein isoforms but may simply be due to excess protein in the nuclear periphery. If this is the case, it is important to understand the mechanisms that regulate protein levels in this region of the cell.

Progerin accumulation not only occurs in HGPS patient cells but has also been detected in cells isolated from healthy individuals across a wide age span (from 1 month to 97 years old) [91,92,93,94]. Progerin levels in older individuals remain lower than those observed in HGPS patients and it is difficult to link these low levels with pathologies. However, aged individuals exhibit numerous cellular features similar to those reported in HGPS children, including irregular nuclear morphology and loss of heterochromatin. Furthermore, overexpression of progerin in normal fibroblasts results in exhibition of HGPS-like phenotypes [93]. These observations add further support to the idea that disruption of cellular function is more likely to be associated with surpassing a threshold of progerin accumulation, rather than the expression of the progerin protein per *se*. In normal individuals, it is unlikely that the presence of a few progerin-containing cells would have any great effect on systemic aging, yet this notion cannot be summarily discounted. Exosomes containing markers of genome instability and senescence including importin, Nesprin-2 and LMNA/C/B1 have been identified [95,96,97]. A few cells exceeding a threshold of progerin may secrete exosomes containing these cargos, to mediate cell–cell communication and promote neighboring cells to senesces or to transport progerin itself. Furthermore, cells that have undergone progerin-induced senescence secrete proinflammatory cytokines (via the senescence associated phenotype (SASP)), promoting further cellular senescence within, for example, subcutaneous white adipose tissue by promoting the senescent cell bystander effects [98].

## 4. Lamina-Associated Protein Accumulation in Neurodegenerative Disease (ND)

**Figure 4:**
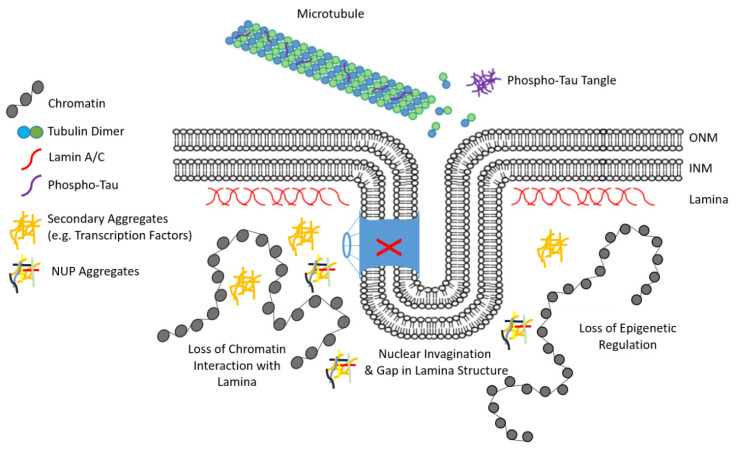
Schematic representation of lamina deformation in neurodegenerative diseases (NDs). Aggregates of proteins, such as phospho-Tau (purple lines) lead to the disruption of nuclear pore complexes (blue structure) and loss of nucleo–cytoplasmic transport (red X). This loss of transport leads to accumulations of nuclear proteins (orange lines) and nuclear pore proteins (NUPs—multicolored lines). The accumulation of these aggregates further disrupts the nuclear lamina, facilitates invagination of the nuclear membranes and, ultimately, the loss of heterochromatin (gray spheres).

Although research on NDs focuses on the impact of protein aggregation within the cytoplasm or extracellular space, there is evidence that protein aggregation impacts nuclear lamina structure and associated processes [82]. The accumulation and aggregation of intracellular phosphorylated-Tau (p-Tau), which normally functions to stabilize microtubules in its non-phosphorylated state, leads to significant disruption of the neuronal nuclear lamina structure as well as significant loss of heterochromatin. In addition, aggregates of p-Tau also impact nuclear pore complexes (NPCs) (Figure 4). NPCs are formed by proteins called nucleoporins (NUPs). In neurons, NPCs exhibit very low levels of exchange and the accumulation of damaged proteins over time. This accumulation is associated with physiological aging as well as NDs [99,100]. In AD caused by p-Tau accumulation, invaginations in the lamina alters NPCs function [82], possibly leading to intra-nuclear inclusion of NUPs [101]. This disruption of NPCs ultimately leads to the accumulation of other protein aggregates through impairment of nucleocytoplasmic transport, including RNA-binding proteins (RBPs) or transcription factors. Nucleo-cytoplasmic transport defects have also been implicated in normal aging [101] and demonstrate another link between lamina protein accumulation in promoting aging and disease. In Amyotrophic Lateral Sclerosis (ALS) and Fronto-Temporal Disease (FTD), the accumulation of the mutated transcription DNA/RNA binding protein-43 (TDP-43) or hyperphosphorylated/ubiquitinated TDP-CTF, result in nuclear membrane deformation and defects in nucleo–cytoplasmic transport through NUP mislocalization [102]. Moreover, TDP-43 aggregates in ALS contain nuclear membrane and NPC proteins and any TDP-43 accumulation increases DNA damage and mislocalization of LINC complex components, such as SUN2 [103], thereby linking protein accumulation in proximity to the nuclear envelope/lamina with disease progression. These observations indicate that protein accumulation within proximity to the nuclear envelope/lamina hastens disease progression. Furthermore, neuronal impairment observed with these diseases implicates abnormal microtubule dynamics with protein accumulation (such as p-Tau), leading to nucleo–cytoplasmic transport impairment and nuclear envelope/lamina disruption [104].

While the description above implies a relationship between the lamina and p-Tau aggregates, the role of the lamina in NDs is likely much more complex. For example, AD-related pathology is associated with a loss of LMNB [82], while knockdown of *LMNB1*, as well as the pore membrane protein of 121 kDa (POM121), increases α-synuclein aggregation in the nuclear envelope in early stages of apoptosis, with potential implications for Parkinson’s disease [105]. In contrast, LMNB is upregulated in both human autopsy tissues and a mouse model of Huntington’s disease [106] and this occurs in a brain region-specific and stage-dependent manner, suggesting a disease-specific mechanism. As mentioned above, LINC complexes influence actin dynamics through RhoA signaling. Actin is ubiquitously expressed and primarily cytoplasmic; however, there is significant actin in the nucleus that forms complexes with cofilin or profilin (targets for RhoA) to form cofilin–actin rods. These rods are critical regulators of gene expression, chromatin architecture and DNA repair processes [107]. Disruption of Ran-GTP/NPC transport can lead to accumulation of actin (as F-actin or G-actin) in both the cytoplasm and nucleus and persistent cofilin–actin rods (cytoplasmic as well as nuclear) have been observed in a number of NDs (e.g., Huntington disease; Alzheimer disease) and also accumulate with age in normal samples [108]. In myopathy, nuclear actin rods correlate with chromatin decondensation as well as defects in nuclear structure and nuclear blebbing [109], parallel to that observed in physiological aging and premature aging syndromes.

Although many of these protein accumulation events resulting in NDs do not localize to the lamina, dysregulation of cellular processes may have knock-on effects that lead to disruption of lamina structure and function. For example, mutations in *presenilin 1* and *presenilin 2* genes (*PSEN-1* and *PSEN-2*), which encode for the PSEN-1 and PSEN-2 core catalytic subunits of the γ-secretase complex, facilitate early onset AD, possibly through apoptosis triggered by the collapse of the nucleus due to lamina disruption [110]. Furthermore, both PSEN-1 [109] and PSEN-2 [110] colocalize with LMNB, and at least with the case of expression of mutant PSEN-2 [110], nuclear collapse is a clear outcome. In addition, huntingtin (HTT) protein containing pathologic polyQ expansions aggregates near the nucleus and disrupts the envelope, while the transgenic R6/2 mouse model of Huntington’s disease displays severe alterations of lamina structure [99,111,112]. These examples of protein aggregation in NDs provide further evidence that many pathologies could be the outcome of disruption of cellular structure and function due to an accumulation of protein, regardless of whether the protein is wildtype, mutated or incorrectly modified. While it remains unclear as to the exact role that these various proteins play (directly or indirectly) in disruption of the lamina and associated nuclear phenotypes, biologic mechanisms have been described, underpinning the need for clarification of the underlying etiologic pathways.

## 5. Mechanisms of Protein Clearance from the Lamina

It is apparent that the accumulation of proteins in the lamina, either mutant or wildtype, leads to the disruption of function and structure resulting in disease and associated with cellular aging. This leads to the questions: What cellular processes or mechanisms are normally involved with clearing these proteins and can we take advantage of these mechanisms to promote health and longevity by maintaining efficient proteostasis?

## 6. Autophagy and Nucleophagy-Mediated Clearance of Lamina Proteins

Autophagy (defined as cellular *self-eating*) is a major conserved ‘recycling’ pathway that plays essential roles in maintaining proteostasis. In this process, extraneous cellular constituents, such as damaged organelles and cytotoxic proteins, are degraded within membrane-bound vesicles called autophagosomes. Following fusion with lysosomes, these cellular constituents are degraded, and their building blocks recycled for energy or protein synthesis [113,114,115]. Loss of autophagy is associated with the initial stages of carcinogenesis, with failure to maintain proteostasis leading to increased cell proliferation [115]. Autophagy further plays important roles in mediating lifespan, with decreased function associated with increased age. This is evident through mutations in *Caenorhabditis elegans* autophagy-specific genes (such as *unc-51, bec-1, atg-7, atg-12* and *atg-18*) causing shortened lifespan [116,117], while overexpression of autophagy genes leads to increased lifespan [118,119,120]. Moreover, overexpression of key transcriptional regulators (such as daf-16/FOXO3a) that promote the transcription of autophagy gene, also results in increased lifespan [121]. The loss of genes encoding autophagy-related proteins, such as *atg-7*, leads to muscle atrophy and reduction in strength as well as decreased rates of myogenic progenitor differentiation [122]. Although muscle and skeletal defects are associated with autophagy-related gene mutations, it is unclear if these phenocopy muscle diseases caused by NETs/lamin-associated protein mutations. Regardless, inhibition of autophagy does result in the accumulation of proteins in the lamina/nuclear envelope [123] in addition to decreased cell viability [124], further linking lamina protein accumulation, autophagy and disease with a failure to maintain proteostasis.

Components of the nucleus can be degraded by a nuclear-specific microautophagy process called nucleophagy. In yeast, nucleophagy occurs in two temporally separate processes: piecemeal nucleophagy and late nucleophagy. Both processes are stimulated by inhibition of nutrient sensing pathways or nitrogen depletion; however, piecemeal nucleophagy is induced under short periods (10–18 h) of nitrogen starvation, whereas late nucleophagy is induced by longer periods (18–24 h) [125,126]. Piecemeal nucleophagy requires direct contact between the nucleus and the lytic vacuole and is mediated by the stepwise assembly of autophagy proteins and pinching off of the nuclear envelope [127,128,129]. Although these pathways are similar, it is unclear if piecemeal nucleophagy and late nucleophagy target different cargos for degradation. The importance of nucleophagy is highlighted by a comprehensive review by Papandreou and colleagues which discusses the role of nucleophagy during pathogenesis involved with several diseases [122]. Further evidence to support the nucleophagy in higher eukaryotes is seen in electron micrographs, which demonstrate the presence of LC3-II-positive vesicles in the perinuclear space of cells with mutations in *LMNA* and *emerin* [124]. A protein that continuously shuttles between the nucleus and the cytoplasm alongside ALFY—p62—and HP1α have also been reported in these perinuclear vesicles [130]. LC3-II is a marker of autophagosomes, and the localization of these vesicles in the perinuclear space is parallel to the placement of nucleophagy vesicles in yeast. In addition, evidence from cells undergoing oncogenesis indicates that nuclear LC3-II interacts with LMNB1 along with other nuclear material targeted for degradation [123]. Although these structures are present, it is unclear why they fail to remove protein accumulations from the lamina resulting in/or as a result of lamina protein accumulation, such as that seen in HGPS or NDs.

## 7. Mechanisms Marking Proteins for Removal from the Lamina

How are lamina-associated proteins targeted for degradation? The autophagy/nucleophagy machinery can access proteins within the lamina. LC3-II is also present in the nucleus and makes contact with LMNB1 to promote its degradation. Although LMNB1 is a long-lived protein in tissues such as the retina [131], loss of LC3-II interaction leads to LMNB1 accumulation concomitantly with cell cycle arrest and senescence [122]. These findings indicate that protein accumulations are not the result of the machinery failing to access their target proteins, a lack of solubility or steric hindrance, but rather a failure of protein degradation. For example, protein accumulations associated with NDs, have been shown to be ubiquitinated, but not cleared while the overexpression of autophagy genes promotes their removal. HGPS fibroblasts also display perinuclear accumulation of autophagosomes with high levels of LC3-II localization [124]; yet, even this does not result in progerin clearance. It is not until autophagy is further induced, through inhibition of pathways such as mTOR [92], that clearance is observed. The mechanisms governing the failure of autophagy and protein degradation machinery to find protein accumulations in the lamina as a result of progerin accumulation or in older cells remains to be elucidated.

An alternative to autophagy is UB-dependent degradation of proteins by the 26S proteasome system. This system involves the covalent attachment of poly-UB chains to target proteins. Tagging of lamina-associated proteins with UB is an important modification for protein turnover [132,133]. An alternative to autophagy is ubiquitin-dependent degradation of proteins by the 26S proteasome system. This system involves the covalent attachment of poly-UB chains to target proteins. Autophagy also utilizes UB; however, target proteins for this pathway are only mono-ubiquitinated. Harhouri and colleagues used cell culture and mouse models of HGPS to demonstrate that MG132-mediated inhibition of the 26S proteasome did not prevent progerin clearance, but actually enhanced it, implicating autophagy as the primary mechanism for lamina protein clearance [134]. Yet, somewhat puzzling was the observation that nuclear export is not required for progerin degradation. This implicates alternative processes, such as those mediated by the formation of p62/LC3-II-containing vesicles, called sequestosomes, may be involved in shuttling cargo to autophagosomes in the perinuclear space/cytoplasm [123].

Although many ubiquitinated lamina-associated proteins are processed and degraded via autophagy, the 26S proteasome also has a role to play in lamina-associated protein degradation. Using mass spectrometry to screen proteins extracted from HEK293 cells, Khanna and colleagues identified RNF123 as one of the E3 ligases responsible for the ubiquitination and subsequent 26S proteasome-mediated degradation, of LMNB1, LAP2a and emerin [133]. RNF123 was also found to interact with LMNA, but unfortunately, this study did not investigate whether LMNA was degraded by autophagy or the 26S proteasome. This appears to contradict other findings indicating autophagy as the primary mechanism for lamina-associated protein degradation [122,134]. Regardless, it is apparent that UB is key in the targeted removal of protein accumulations. In support of this, Borroni and colleagues identified the E3 UB-ligase, SMAD-specific E3 UB protein ligase (SMURF2) as a direct interaction partner of both LMNA and progerin in HGPS cells. Overexpression of SMURF2 decreased progerin levels and improved HGPS phenotypes [135,136]. Observations that ubiquitination stimulates both pathways indicates there may be a complex interplay between autophagy and the 26S proteasome in mediating proteostasis of the lamina.

In addition to its interaction with LMNA, SMURF2 physically interacts with the Anaphase Promoting Complex (APC) [137,138,139], a nuclear E3 UB-ligase that mediates the degradation of targets involved in cell cycle regulation, DNA replication, genome stability, nutrient sensing, cell differentiation and cancer [140,141,142,143]. This physical interaction indicates that the APC may have roles in the clearance of proteins from the lamina. The APC is a 19-subunit complex containing 14 different proteins [142,144,145], including the coactivators CDC20 (Cell division cycle 20; required for mitotic progression) or CDH1 (CDC20 homolog 1; required for mitotic exit and G1 maintenance). APC function is associated with regulating yeast lifespan [146] and APC-Cdh1-deficient mouse embryonic fibroblasts undergo replicative senescence due to the accumulation of APC substrates [147]. APC has also been linked with the clearance of the inner nuclear membrane protein Mps3 [148]. Furthermore, AD-related pathology can also result from APC dysregulation when cyclin B accumulates and induces aberrant cell cycle entry, promoting cell death [149]. This example, as well as several others discussed elsewhere, link APC function to protein accumulation, disease and aging [150,151].

## 8. Manipulating for Lamina Protein Clearance

For protein accumulation diseases such as the laminopathies, HGPS and NDs, removal of the cytotoxic proteins through genetic manipulation would provide cures. The hope for the future is the use of novel technologies, such as CRISPR-Cas9-mediated genome editing [152,153,154], to correct these genetic defects by, for example, silencing the dominant-negative allele of *LMNA* or upregulating autophagy genes. HGPS mouse models edited by CRISPR-Cas9 have shown the potential of this. CRISPR-Cas9 has also been used to correct defects observed in AD models harboring mutations in *PSEN-2* [155]. Although removing the mutation causing HGPS by somatic CRISPR-Cas9 mediated genome editing could in theory provide a cure for these diseases, efficiently targeting all or even a reasonable number of cells within a juvenile or adult organism has not been achievable and, thus, genome editing approaches will not be available within the foreseeable future. As such, alternative strategies are required for the treatment of these diseases. One viable option is the targeting of protein accumulations for degradation. Research linking the role of autophagy’s impact on cellular and organismal lifespan as well as cellular nutrient and stress-sensing pathways has burgeoned in the last decade. This research has identified a growing list of pharmacological agents as well as diet and dietary compounds that lead to increased autophagy function and clearance of excess cellular protein [156,157,158,159,160,161,162,163,164,165,166,167].

**Figure 5:**
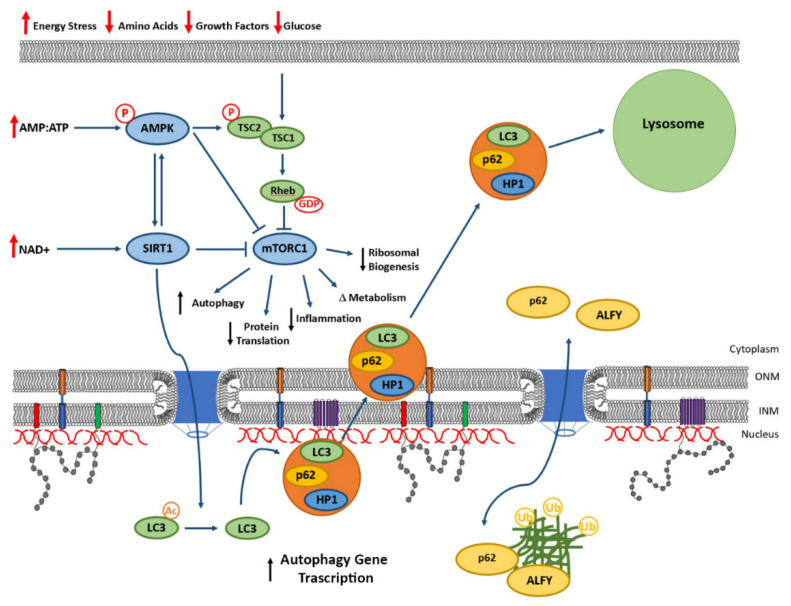
Linking lamina protein levels with cellular energy sensing. Schematic representation of the link between cellular energy sensing pathways (Sirtuin1 deacetylase (SIRT1) and adenosine mono-phosphate kinase (AMPK)) and mammalian target of rapamycin complex 1 (mTORC1). Increased levels of NAD+ and AMP upregulate SIRT1 and AMPK, respectively leading to downregulation of mTORC1 function. Decreases in growth factors and amino acids also downregulate mTORC1 function. Downstream functions of mTORC1 are indicated. SIRT1 interactions with LC3 are also indicated, leading to the formation of LC3, p62 and HP1 containing vesicles carrying cargo to lysosomes. This also leads to p62 and ALFY binding ubiquitinated cargo for nuclear export.

One possible target is the mammalian target of rapamycin complex 1 (mTORC1), which is tightly linked to autophagy function. Under conditions of growth factor stimulation or high levels of cellular nutrients (including glucose and amino acids) (Figure 5), mTORC1 is in an active state and promotes cellular proliferation and protein translation, in addition to repressing autophagy [168,169,170,171]. In the absence of these growth factors and cellular stress, such as low energy availability, mTORC1 function is repressed. Key in modulating mTORC1 function in response to cellular energy status are adenosine mono-phosphate kinase (AMPK) and the Sirtuin1 deacetylase (SIRT1). AMPK is active in response to increased AMP:ATP ratios and SIRT1 is activated by high cellular levels of NAD^+^. Although the theme through this review has been that too much protein generally has negative impacts, overexpression of both SIRT1 and AMPK represses mTORC1 and upregulates autophagy, leading to increased cellular and organismal lifespan in multiple systems and organisms [172,173,174]. Furthermore, activation of SIRT1 results in deacetylation of nuclear LC3, enabling relocalization of LC3 to the cytoplasm from the nucleus and promoting autophagy and potentially formation of nucleophagy-associated vesicles. Although there is debate surrounding target specificity of many compounds that target nutrient/energy sensing pathways, there is strong evidence linking increased AMPK and SIRT1 function with compounds such as metformin [158,159,162,175,176,177] and resveratrol [160,178,179,180,181,182,183], respectively to increased health and lifespan. The benefits of these compounds is likely through increased autophagy and indicates the potential therapeutic use of these compounds (or similar molecules with higher target specificity) to promote protein accumulation clearance from the lamina. Upregulation of AMPK with metformin or SIRT1 with resveratrol has been demonstrated to exert beneficial effects in NDs. For example, metformin significantly reduces the levels of phosphorylated α-synuclein, a modified form of the protein that is more frequently present in Parkinson’s disease (PD) [184,185], whereas resveratrol promotes the clearance of β-amyloid protein, the core component of the senile plaque found in AD, in HEK293 cells expressing the amyloid precursor protein [186], thus demonstrating the efficacy of this approach. Induction of autophagy can also be accomplished using compounds that directly inhibit mTORC1 function. Rapamycin, the compound initially used to identify mTORC1, is bound by the catalytic TOR and FKBP12 subunits of mTORC1, resulting in decreased function and upregulation of autophagy. Cao and colleagues demonstrated that rapamycin treatment leads to increased rates of progerin degradation, improved nuclear morphology and increased cellular lifespan, which paralleled the degradation of progerin following AMPK activation via metformin [187]. The enhancement of autophagy via rapamycin has also been found to be beneficial in NDs. In MPTP-treated mice, a model for PD, rapamycin treatment reduced dopaminergic cell death [188]. Moreover, when mice modeling HD are treated with the rapamycin ester temsirolimus, there is a decreased aggregation of HTT protein [189], indicating the beneficial effect that rapamycin can provide in these pathologies. In addition, a new generation of rapamycin analogs, called rapalogues (such as Everolimus) [190] exhibit higher specificity with fewer side-effects than rapamycin. Although there are concerns over impacts of immune responsiveness and potential negative effects in the elderly, a growing body of evidence for the benefits of managing cellular energy and stress-sensing pathways through those agents described above is growing and providing tangible strategies to increase health through the clearance of protein accumulations of the nuclear lamina via autophagy.

## 9. Concluding Remarks

Numerous examples demonstrate that maintaining proteostasis is essential for cell function and health. The failure to clear any accumulation of wildtype proteins or the accumulation and/or deposition of cytotoxic variants impacts the integrity and function of the nuclear lamina, and leads to the disruption of essential cell and genome functions. Increasing our understanding of the functional consequences of protein accumulation within this structure is certainly important, but as important—or perhaps more so—is to fully understand how the mechanisms that increase protein clearance can be specifically targeted and manipulated so as to intervene in diseases of protein accumulation/dysfunctional proteostasis, ranging from cancers to neurodegenerative disorders and to potentially slow the aging process itself.

## Abbreviations

AD	Alzheimer’s Disease
AMPK	Adenosine mono-phosphate kinase
ALS	Amyotrophic Lateral Sclerosis
APC	Anaphase Promoting Complex
ATG	Autophagy related gene
Cdc20	Cell division cycle 20
CDH1	CDC20 homolog 1
DNMT	DNA methyl transferases
FTD	Fronto-Temporal Disease
HP1a	Heterochromatin Protein 1a
HGPS	Hutchinson–Gilford Progeria Syndrome
INM	Inner nuclear membrane
LMN	Lamin
LMNA	Lamin A
LMNB1	Lamin B1
LMNB2	Lamin B2
LMNC	Lamin C
LBR	Lamin B receptor
LADs	Lamina Associated Domains
LAP	Lamina associated peptide
LC3	Light Chain 3
LC3-II	Light Chain 3 isoform II
LINC	Linker of nucleoplasm to cytoplasm complex
TADs	Topologically associated domains
nesprins	Nuclear envelope spectrin-repeat proteins
KASH	Klarsicht/ANC1/Syne-1 homology
mTORC1	Mammalian target of rapamycin complex 1
NDs	Neurodegenerative diseases
NUPs	Nucleoporins
NPC	Nuclear pore complexes
NETs	Nuclear envelop transmembrane proteins
NUPs	Nuclear pore proteins
ONM	Outer nuclear membrane
PD	Parkinson’s disease
-POM121	Pore membrane protein of 121 kDa
PSEN1	Presenilin 1
PSEN2	Presenilin 2
HTT	Huntingtin protein
SUN	SAD1/UNC-84 homology
SASP	Senescence associated phenotype
SIRT1	Sirtuin1
SMURF2	SMAD-specific E3 UB protein ligase
UB	Ubiquitin
ZMPSTE24	Zinc Metallo peptidase STE24
